# Trajectory Tracking within a Hierarchical Primitive-Based Learning Approach

**DOI:** 10.3390/e24070889

**Published:** 2022-06-28

**Authors:** Mircea-Bogdan Radac

**Affiliations:** Department of Automation and Applied Informatics, Politehnica University of Timisoara, 300223 Timisoara, Romania; mircea.radac@upt.ro

**Keywords:** virtual state, model reference tracking, temperature control system, electrical braking system, approximate dynamic programming, neural networks, optimal control, reinforcement learning, state feedback control, primitives, iterative learning control, data-driven, model-free, hierarchical control

## Abstract

A hierarchical learning control framework (HLF) has been validated on two affordable control laboratories: an active temperature control system (ATCS) and an electrical rheostatic braking system (EBS). The proposed HLF is data-driven and model-free, while being applicable on general control tracking tasks which are omnipresent. At the lowermost level, L1, virtual state-feedback control is learned from input–output data, using a recently proposed virtual state-feedback reference tuning (VSFRT) principle. L1 ensures a linear reference model tracking (or matching) and thus, indirect closed-loop control system (CLCS) linearization. On top of L1, an experiment-driven model-free iterative learning control (EDMFILC) is then applied for learning reference input–controlled outputs pairs, coined as primitives. The primitives’ signals at the L2 level encode the CLCS dynamics, which are not explicitly used in the learning phase. Data reusability is applied to derive monotonic and safely guaranteed learning convergence. The learning primitives in the L2 level are finally used in the uppermost and final L3 level, where a decomposition/recomposition operation enables prediction of the optimal reference input assuring optimal tracking of a previously unseen trajectory, without relearning by repetitions, as it was in level L2. Hence, the HLF enables control systems to generalize their tracking behavior to new scenarios by extrapolating their current knowledge base. The proposed HLF framework endows the CLCSs with learning, memorization and generalization features which are specific to intelligent organisms. This may be considered as an advancement towards intelligent, generalizable and adaptive control systems.

## 1. Introduction

A hierarchical primitive-based learning framework (HLF) for trajectory tracking has been proposed and extended recently in [1,2,3]. Its main goal is to make the control systems (CSs) capable of extending a current knowledge base of scenarios (or experiences) consisting of different, memorized tracking tasks, towards new tracking tasks that have not been seen before. While the knowledge base of tracking tasks is improved repetitively in a trial- or iterative-based manner with respect to an optimality criterion, it is required that for new tracking tasks, the unseen-before trajectory is to be optimally tracked without giving the chance of improvement by repetitions. Therefore, the problem is one where the CS is required to extrapolate its current knowledge base to new, unseen-before scenarios. It is a form of generalization ability which is specific to living organisms and can be regarded as a form of intelligence under the name of cognitive control.

The means to achieve such generalization proposes an HLF approach [3]: first, the lower level L1 is dedicated to learning output- or state-feedback controllers for the underlying nonlinear system with unknown dynamics. Thus, this is a form of model-free or data-driven control. The L1 learning aims for ensuring that the closed-loop CS (CLCS) matches a linear reference model, in response to a given reference input; in addition to adding stability, uncertainty robustness and disturbance rejection to the CLCS, it enables a linear closed-loop behavior which fits the linear superposition principle. The latter allows for straightforward time and amplitude scaling of the tracking tasks. Moreover, the L1 level leaves open the possibility of applying a secondary, intermediate learning level L2, over a linear assumption about the CLCS. L2 learning allows for iterative tracking improvement by means of trials/iterations/repetitions, which is the well-known approach of the iterative learning control (ILC) framework. The CLCS’s {reference inputs, controlled outputs} pairs were coined as primitive pairs, or simply, *primitives*. The principal aspect about the primitives is that they indirectly encode the reference input-controlled output CLCS dynamics (or behavior). They are useable in a tertiary (and final, uppermost) L3 learning level, to optimally predict the best reference input which drives the CLCS’s output towards tracking a novel desired trajectory; this time without having to re-learn by trials.

Level-wise, it is desirable that the learning in all levels takes place without using explicit mathematical models [3]. Then, the framework’s generalization resembles intelligent organisms who do not explicitly solve mathematical equations in their brains in order to enable such features (intuitively, we think here about the neuro-muscular control behavior and not about conscience-based cognitive processes developed by humans, which may involve different abstract representations such as models). The hierarchical primitive-based framework is detailed as follows.

At level L1, a nonlinear observability property invoked for the underlying controlled system allows for an equivalent virtual state representation constructed from present and past input–output data samples. This ultimately renders a virtual state–space transformation of the original unknown input–output dynamics system, where the virtual states are fully measurable. This “state” is useable for virtual state-feedback control learning, to ensure many control objectives, among which the linear model-reference matching (or tracking) is very popular. Two compatible approaches have recently been proposed in this context: virtual state-feedback reference tuning (VSFRT) [4] and model-free value iteration reinforcement learning (MFVIRL) [3,4,5]. These two approaches share the same goal of model-reference matching; however, they have very different methodologies and also some different traits: VSFRT is “one-shot” non-iterative in the learning phase, whereas MFVIRL is iterative throughout its learning phase. Both are capable of learning (virtual) state-feedback control, over linearly or nonlinearly parameterized controllers, and over linear or nonlinear unknown dynamical systems. This is a form of implicit model-free feedback linearization where the virtual state-feedback controller learns to cancel the controlled system nonlinearities in order to make the CLCS behave linearly from the refence input to the controlled output. Some recent applications with stability and convergence guarantees for these two techniques are mentioned in [4] for VSFRT and in [5,6] for MFVIRL. We keep in mind that VSFRT stems from the original popular VRFT approach in control systems [7,8,9,10,11,12], whereas MFVIRL is a reinforcement Q-learning approach from the well-known reinforcement learning framework [13,14,15,16,17,18], which is common both with artificial intelligence research [19,20,21,22] and with classical control with a focus on theoretical research [23,24,25,26,27,28,29,30] and applications [31,32,33,34,35,36].

The L2 level learning process relies on the CLCS linearity, allowing for the application of one variant of ILC which is agnostic to the CLCS dynamics, called the experiment-driven model-free ILC (EDMFILC) [1,2,3]. This technique belongs to the popular data-driven ILC approaches [37,38,39,40,41,42] as part of data-driven research [43,44,45,46,47]. Here, the convergence analysis selects a conservative learning gain, based on equivalent CLCS models resulting from the actual reference model and from identified models based on the reusable input–output data.

The L3 level learning brings the idea of motion primitives from robotics towards generalized tracking behavior. There are many known approaches to primitive-based tracking control, both older and more recent [48,49,50,51,52]. Their taxonomy is not studied here because it has been reviewed elsewhere, e.g., in [1,2,3]. Most of these approaches rely on learning control principles in various settings, ranging from security-enabled control system [53,54] to learning control in uncertain environments [55,56] and even iterative learning approach to model predictive control [57]. In the proposed HLF, the primitives are copied, extended, delayed and padded for use, according to the linearity superposition principle, to predict the optimal reference input. It is therefore the superposition principle which ultimately grants the CLCS’s generalization ability.

Some appealing features of the primitive-based HLF are enumerated:

- Capability to deal with multivariable MIMO systems in level L1 and with MIMO CLCS in level L2 was proven in previous studies.
- Theoretical convergence and stability guarantee at all learning levels, via different mechanisms. This is based on common data-driven assumptions in level L1, on data reusability at level L2 and on approximation error boundedness assumptions in level L3.
- Ability to deal with desired trajectories of varying length at level L3.
- Ability to handle inequality-type, amplitude- and rate-constraints on the output trajectory indirectly in a soft-wise style, by desired trajectory clipping at level L3 and by good linear model reference matching in level L1.
- Displaying intelligent reasoning based on memorization, learning, feedback used on different levels, adaptability and robustness and generalization from previously accumulated experience to infer optimal behavior towards new unseen tasks. These traits make the framework cognitive-based.

The HLF has been validated on a number of complex nonlinear mono- and multivariable systems such as: an aerodynamic system [4], robotic arm [2], electrical voltage control [1,3]. This paper’s goal is to prove the framework’s applicability and effectiveness on other applications which are very different in nature: the active temperature control system (ATCS) and the electrical braking system (EBS). Both ATCS and EBS have wide industrial occurrence; therefore, they impact many potential applications. Hopefully, this will elucidate more about the framework’s generalization ability and bring the CSs a step closer to the desirable features of intelligent control: learnability, adaptability, generalization and robustness in harsh environments. The realistic experimental validation on hardware shows that the HLF’s intermediate levels exhibit robustness against noise, against the CLCS’s approximate linear behavior and against the varying desired trajectory’s settings such as length and constraints. As a secondary objective, the proposed HLF shows that modern machine learning methods (supervised learning in particular) leverage control system techniques to reach capabilities beyond their classical scope. To this end, a long short-term memory (LSTM) nonlinear recurrent neural network (NN) controller was used for the first time with the VSFRT approach. The resulting nonlinear controller showed superior behavior with respect to a plain feedforward NN controller, which was trained with the same VSFRT principle. The explanation lies with the LSTM’s ability to learn longer-term dependencies for time sequences. Function approximation theory is again employed in the third level (level L3) for predicting optimized reference inputs in the context of dynamical systems.

This paper discusses basic theoretical assumptions about the controlled systems and introduces the model reference control problem in Section 2. Application of the proposed primitive-based learning framework to the ATCS is detailed in Section 3, whereas the application to the EBS is presented in Section 4. Concluding remarks are outlined in Section 5.

## 2. Model Reference Control with Virtual State-Feedback

### 2.1. The Unknown Dynamic System Observability

The nonlinear unknown controlled system has the input–output discrete-time description (*k* indexes time sample):

(1) $$y_{k}=f\left(y_{k-1},\ldots ,y_{k-ny},u_{k-1},\ldots ,u_{k-nu}\right),$$

fulfilling the following assumptions:

A1. The input $u_{k}={\left[u_{k,1},\ldots ,u_{k,m_{u}}\right]}^{T}\in \Omega_{U}\subset \;{\mathbb{R}}^{m_{u}}$ has known domain $\Omega_{U}$, and the output $y_{k}={\left[y_{k,1},\ldots ,y_{k,m_{y}}\right]}^{T}\in \Omega_{Y}\subset \;{\mathbb{R}}^{m_{y}}$ has known domain $\Omega_{Y}.$

A2. The positive orders $n_{y},\;n_{u}$ are unknown integers.

A3. The nonlinear map $f:\Omega_{Y}\times \ldots \times \Omega_{Y}\times \Omega_{U}\times \ldots \times \Omega_{U}\to \Omega_{Y}$ is continuously differentiable and unknown.

A4. (1) has an equivalent minimal state–space nonlinear realization.

(2) $$\{\begin{array}{c} \;\sigma_{k+1}=g\left(\sigma_{k},u_{k}\right),\\ \;y_{k}=h\left(\sigma_{k}\right), \end{array}$$

where $\sigma_{k}={\left[\sigma_{k,1}\ldots \sigma_{k,n}\right]}^{T}\in \Omega_{\Sigma }\subset {\mathbb{R}}^{n}$ is the system’s state of unknown order n and unknown domain $\Omega_{\Sigma }$, which is again unmeasured.

A5. The nonlinear system (1) is input–output-controllable and the pair (g,h) is observable.

**Definition** **1** [3]. *The unknown observability index of (1) is the minimal value $\tau_{min}$* *of τ* *for which state $\sigma_{k}$* *is fully recoverable from the I/O measurement vectors $Y_{\bar{k,k-\tau }}={\left[{\left(y_{k}\right)}^{T}\ldots \;{\left(y_{k-\tau }\right)}^{T}\right]}^{T},\;U_{\bar{k-1,k-\tau }}=[{\left(u_{k-1}\right)}^{T},\ldots ,$* ${\left(u_{k-\tau }\right)}^{T}{]}^{T}$*. This index has the same role as with observable linear systems.*

**Theorem** **1.** *There exists a virtual state–space representation.*

(3) $$\{\begin{array}{c} \;s_{k+1}=\bar{g}\left(s_{k},u_{k}\right),\\ \;y_{k}=s_{k,1}, \end{array}\;$$

*where *$\bar{g}$ *is a partially unknown system function and *$s_{k}={\left[{\left(Y_{\bar{k,k-\tau }}\right)}^{T},\;{\left(U_{\bar{k-1,k-\tau }}\right)}^{T}\right]}^{T}\;\overset{\Delta }{=}\;{\left[{\left(s_{k,1}\right)}^{T},\;{\left(s_{k,2}\right)}^{T},\;\ldots ,\;{\left(s_{k,2\tau +1}\right)}^{T}\right]}^{T}\in \underset{\tau +1\;times}{\underbrace{\Omega_{Y}\times \ldots \times \Omega_{Y}}}\times \underset{\tau \;times}{\underbrace{\Omega_{U}\times \ldots \times \Omega_{U}}}≜$$\Omega_{S}\subset {\mathbb{R}}^{m_{y}\left(\tau +1\right)+m_{u}\tau }$*is called the virtual state from whose definition it clearly results that *$s_{k,1}=y_{k},\ldots ,s_{k,2\tau +1}=u_{k-\tau }$*. Additionally, *$s_{k}$*is an alias for *$\sigma_{k}$*from another dimension/space, being related through an unknown transformation *$\sigma_{k}=T\left(s_{k}\right)$.

**Proof of Theorem** **1** . Proof is based on Theorem 1 from [6] using assumptions A1–A5.

*Observation* 1. The virtual state–space (3) is fully state-measurable.

*Observation* 2. The virtual state–space model (3) is input–output-controllable and has the same input–output behavior of (1) and (2).

*Observation* 3. Input delays in (1) can still lead to transformations (3) by the appropriate introduction of additional states. Time delay affects the relative degree of the basic system (1) and can be measured from input–output data. To accommodate this case, another assumption follows.

A6. The system’s (1) relative degree is known.

For the subsequent output model reference tracking design, the minimum-phase assumption about the system (1) is also enforced. The motivation is that the non-minimum-phase behavior is more troublesome to handle within the model reference control with unknown system dynamics.

*Observation* 4. The size of $s_{k}$ built from input–output historical data can be much greater than the size of the true state vector $\sigma_{k}$. Dimensionality reduction techniques specific to machine learning, such as principal component analysis (PCA) or autoencoders (AEs) are employed to retain the relevant transformed features emerging from the virtual state $s_{k}$ [4]  □.

### 2.2. The Reference Model

A linear, strictly causal reference model described in state–space form is presented as

(4) $$\{\begin{array}{c} \;s_{k+1}^{RM}=As_{k}^{RM}+B\rho_{k},\\ \;y_{k}^{RM}=Cs_{k}^{RM}, \end{array}$$

where $s_{k}^{RM}={\left[s_{k,1}^{RM},\ldots ,s_{k,n_{m}}^{RM}\right]}^{T}\in \;\Omega_{S_{RM}}\;\subset {\mathbb{R}}^{n_{m}}$ is the $n_{m}$-dimensional reference model state, $\rho_{k}={\left[\rho_{k,1},\ldots ,\rho_{k,m_{y}}\right]}^{T}\in \Omega_{\rho }\subset \;{\mathbb{R}}^{m_{y}}$ simultaneously excites the reference model and the CLCS and there is a one-to-one relationship between the components of $\rho_{k},y_{k},y_{k}^{RM}$, where $y_{k}^{RM}={\left[y_{k,1}^{RM},\ldots ,\;y_{k,m_{y}}^{RM}\right]}^{T}\in \;\Omega_{Y_{RM}}\subset \;{\mathbb{R}}^{m_{y}}$: each component of $\rho_{k}$ drives a corresponding component from $y_{k}$ and $y_{k}^{RM}$, respectively. Assuming an input–output pulse transfer matrix $y_{k}^{RM}=M\left(q\right)\rho_{k}$, $q^{-1}$ is the one step delay operator operating on discrete-time signals.

For the model reference control, M(q) must carefully consider the non-minimum-phase behavior of (1) together with its relative degree and bandwidth. These are classical requirements for the model reference control problem where the controller tuning for the nonlinear system (1) should make its output $y_{k}$ track $y_{k}^{RM}$ when both the CLCS and the reference model are excited by $\rho_{k}$. M(q) is mostly diagonal, to obtain decoupled control channels.

### 2.3. The Model Reference Control

The model reference control tracking problem can formally be written as the optimal infinite-horizon control [3]

(5) $$u_{k}^{*}=arg\underset{u_{k}}{min}V_{RM}^{\infty }\left(u_{k}\right),\;V_{RM}^{\infty }\left(u_{k}\right)=\sum_{k=0}^{\infty }\left|\left|y_{k}\left(u_{k}\right)-y_{k}^{RM}\right|\right|{}_{2}^{2},\;\;s.t.\;dynamics\;\left(3\right)\;\left(or\;\left(1\right)\right)+\left(4\right).$$

In (5), $V_{RM}^{\infty }$ is the cost function measuring the deviation of the CLCS output from that of the reference model output. The closed-form VSFRT solution to (5) is expressed as $u_{k}=C\left(s_{k}^{ext}\right)$, with C(.) being a linear/nonlinear map over an extended state comprising of $s_{k}^{ext}={\left[s_{k}^{T},\rho_{k}^{T}\right]}^{T}$. Both $s_{k}$ and $\rho_{k}$ will be replaced by their offline calculated counterparts ${\tilde{s}}_{k}$ and ${\tilde{\rho }}_{k}$ following the VSFRT principle. Problem (5) is indirectly solved as the next equivalent controller identification problem [3,4]

(6) $$\pi^{*}=arg\underset{\pi }{min}V_{VR}^{N}\left(\pi \right),\;V_{VR}^{N}\left(\pi \right)=\frac{1}{N}\overset{N}{\underset{k=1}{\sum }}{\left|\left|u_{k}-C\left(s_{k}^{ext},\pi \right)\right|\right|}^{2},$$

where π is the controller function parameter leading to notation $C\left(s_{k}^{ext},\pi \right)$ (here, the controller can be an NN or other type of approximator) [4,5]. In [4,5], it was motivated why the reference model state $s_{k}^{RM}$ should not be included within $s_{k}^{ext}$ because the former correlates with $\rho_{k}$. Additionally, [4] proposed theoretical stability analysis of the CLCS with the resulting controller and how the $V_{VR}^{N}$ from (6) and $V_{RM}^{\infty }$ from (5) are related. For other solutions to the model reference tracking problem (5), such as reinforcement learning, a different $s_{k}^{ext}$ is required in order to ensure the MDP assumptions about the controlled process [1,3,4,5,6].

**Figure 1:**
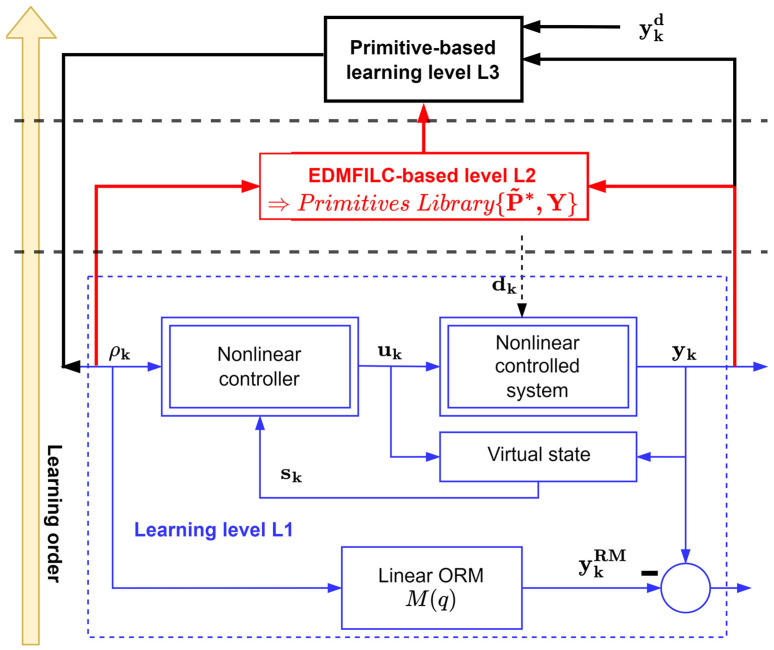
The three-levelled HLF.

After solving the model reference control problem at level L1, learning level L2 takes place, using the EDMFILC strategy. The intention is to learn the primitive pairs in this level and use them to populate the primitive’s library. The proposed three-levelled HLF is completed with the final level, L3. Here, the primitive outputs of the learned pairs are used for decomposing the desired new trajectory, whereas the primitive inputs are used to recompose the optimized reference input [1,2,3]. The HLF architecture is captured in the diagram in Figure 1.

## 3. The Active Temperature Control System

### 3.1. System Description

**Figure 2:**
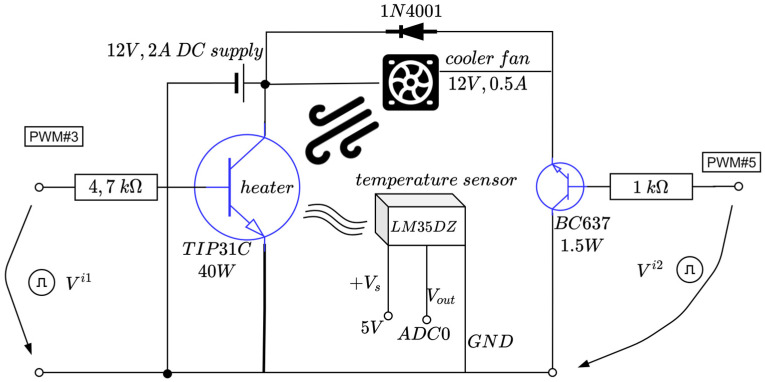
Schematic diagram of the ATCS (adapted from [58]).

**Figure 3:**
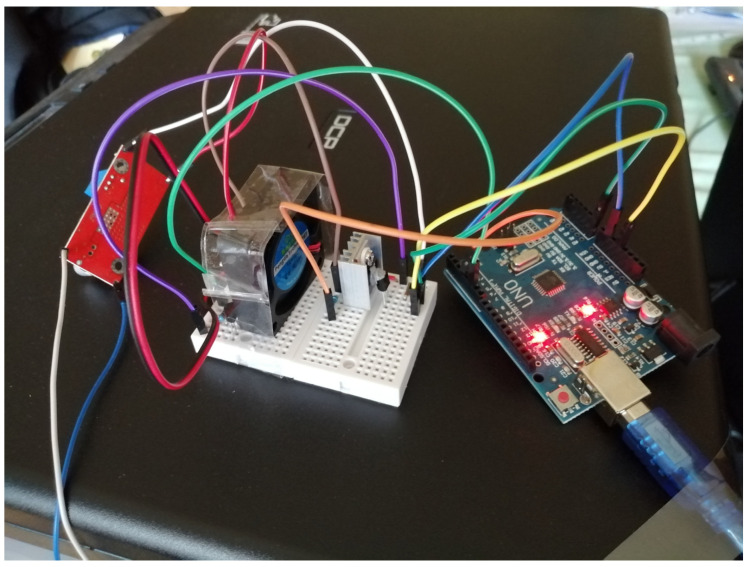
Illustration of the realized ATCS hardware.

The active temperature control system (ACTS) is an Arduino-centered device dedicated to temperature control in a room-controlled temperature environment [58]. It has an active heating module in terms of a TIP31C power transistor. Additionally, an active cooler in terms of a fan relying on a DC motor with nominal characteristic consumptions of 0.5 amps (A) at about 120 revolutions per second. Using an analogue temperature measuring sensor based on LM35DZ, the main power transistor’s temperature is read and used for feedback control. The equipment is small in scale and is depicted in Figure 2 and Figure 3. A single power supply of 12 volts and maximum 2 amps is used from a commercially available DC–DC buck–boost converter. The power supply alternatively drives the power transistor and the DC fan via control logic: only one element is active at a time. Both elements are driven by pulse width modulation (PWM).

The fan control circuit uses a 1N4001 protection diode and a BC637 (up to 1.5 watts) transistor which allows for varying fan speed, thus accelerating the cooling process by heat dissipation (which otherwise would be a slow process given the system’s nature).

The heater is the power transistor itself, capable of a maximum 40 watts and gradually controlling its temperature through the PWM switching logic. A heatsink is attached to the TIP31C’s body to better dissipate heat, while the LM35DZ temperature sensor is physically connected to the power transistor using thermal paste for better heat transfer.

A sampling time $T_{s}$ of 20 s is sufficient to capture the ATCS’s dynamics. The TIP31C’s surface temperature is measured as the voltage $V_{out}$ of the analogue sensor and then converted via the Arduino’s ADC port #0. Finally, the controlled output $y_{k}\left[{}^{\circ}C/100\right]$ is just the normalized equivalent temperature in degrees Celsius divided by 100 and used for feedback control. The control to the heater and cooler transistors uses the PWM output ports (herein, ports #3 and #5) from Arduino. The alternating high-level switching logic activating the cooler/heater (just one at a time) uses the equation [58]

(7) $$\{\begin{array}{c} V^{i1}=max\left(min\left(0.2+\left|u\right|,1\right),0\right)\times 5V,V^{i2}=0V,\;when\;u\geq 0,\\ V^{i1}=0V,V^{i2}=max\left(min\left(0.15+\left|u\right|,1\right),0\right)\times 5V,\;when\;u<0. \end{array}$$

In the above equation, $V^{i1}$ and $V^{i2}$ are the voltages controlling the heater and the cooler, respectively (see Figure 3), whereas the thresholds 0.15 and 0.2 compensate the dead-zones in the cooler and heater, respectively. The TIPC31C does not drive the current below 1 V (hence, no heat is produced) and the fan DC motor does not spin for a voltage supply under 0.75 V. Equation (2) ensures proper saturation of the voltages per *min*, *max* functions. The term u from (2) represents the signed a-dimensional control input $u_{k}\in \left[-1;1\right]$, which interprets a duty cycle of the two PWMs, with the sign ensuring alternate functioning of the heater and the cooler, respectively.

Next, the input–output data collection step, intended for the virtual state feedback control learning process, is unveiled.

### 3.2. ATCS Input–Output Data Collection for Learning Low-Level L1 Control Dedicated to Model Reference Tracking

**Figure 4:**
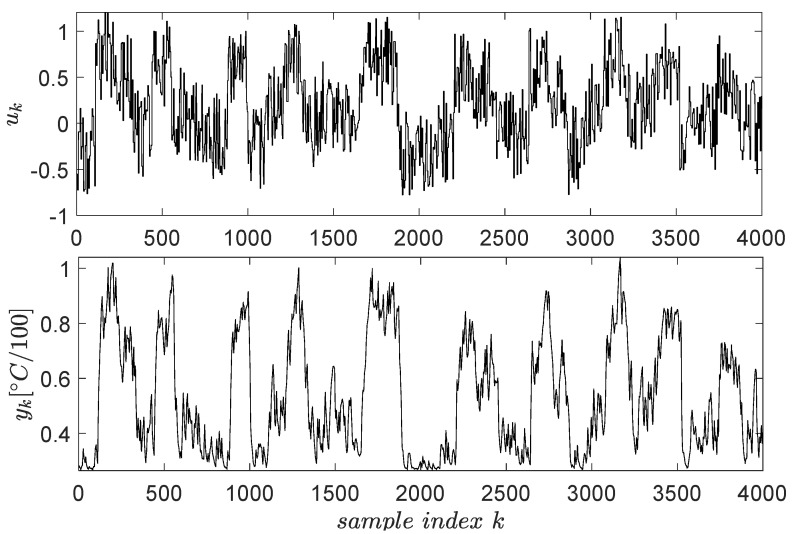
Input–output data collected from the ATCS.

The open-loop input–output data collection uses a signal $u_{k}$ described as piece-wise constant whose levels are randomly distributed in the range [−0.3;0.8]. The switching period of these levels is 2200 s (the system has high inertia, being a thermal process). To capture all relevant dynamics of the system, the exploration is stimulated by an additive noise similarly modelled as the base signal. The noise levels were uniformly distributed in [−0.5;0.5] and its switching period being 100 s. The resulting input–output data are presented in Figure 4 for N=4000 samples.

To learn a model-free controller using the collected input–output data, the VSFRT procedure is applied, as thoroughly described in [2,4,58]. The VSFRT paradigm ensures the procedure for designing a linear (or nonlinear) virtual state-feedback controller which matches the closed-loop control system to a reference model.

First, a reference model is selected as M(s)=1/(500s+1) (s is the continuous-time transfer function Laplace domain operator). Its selection is qualitative, based on several key observations: the ATCS is highly damped, it has no dead-time either with respect to the data acquisition process nor with its intrinsic dynamics, its bandwidth is matched with the natural open-loop ATCS’s bandwidth, being slightly higher (faster response on closed-loop than in open-loop which is common sense for the control). This refence model is discretized using zero-order hold for a sampling interval of 20 s, to render the discrete-time filter M(q). The fact that M(q) is linear indirectly requires the virtual state-feedback controller to render a linear CLCS over the ATCS.

Then, the steps below are followed, in order:

*Step* 1. Define the observability index τ=2, and form the trajectory $\left\{u_{k},\;y_{k},{\tilde{s}}_{k}\right\}$, where the virtual state ${\tilde{s}}_{k}=\left[y_{k},\;y_{k-1},\;y_{k-2},\;u_{k-1},\;u_{k-2}\right]$ is built by assuming that the nonlinear ATCS is observable. Using the discrete time index $k=\bar{1,N}$, a total number of 3998 tuples of the form $\left\{u_{k},\;y_{k},{\tilde{s}}_{k}\right\}$ are obtained.

*Step* 2. The virtual reference input is computed as ${\tilde{\rho }}_{k}=M^{-1}\left(q\right)y_{k}^{f}$, where $y_{k}^{f}$ is the low-pass-filtered version of $y_{k}$ through the filter $\frac{0.45}{1-0.55q^{-1}}$, because $y_{k}$ is slightly noisy. Notably, $M^{-1}\left(q\right)$ involves a non-causal filtering operation which is not problematic because it is performed offline.

*Step* 3. Construct the regressor state as $s_{k}^{ext}={\left[{\tilde{s}}_{k}^{T},\;1,\;{\tilde{\rho }}_{k}\right]}^{T},1\leq k\leq 3998$. The constant “1” is added into the regressor to allow for the offset coefficient identification, leading to a linear affine virtual state-feedback controller.

*Step* 4. Parameterize the virtual state-feedback controller in a linear fashion, as $u_{k}=K^{T}s_{k}^{ext}$. The VSFRT goal is to achieve model reference matching by indirectly solving the controller identification problem [2,4,53]

(8) $$K^{*}=arg\underset{K}{min}V_{VR}^{N}\left(K\right),\;V_{VR}^{N}\left(K\right)=\frac{1}{N}\overset{N}{\underset{k=1}{\sum }}{\left|\left|u_{k}-K^{T}s_{k}^{ext}\right|\right|}^{2}.$$

*Step* 5. The problem (8) is posed as an overdetermined linear system of equations

(9) $$\left[\begin{array}{c} {\left(s_{1}^{ext}\right)}^{T}\\ \ldots \\ {\left(s_{N}^{ext}\right)}^{T} \end{array}\right]K=\left[\begin{array}{c} u_{1}\\ \ldots \\ u_{N} \end{array}\right]\Leftrightarrow M_{1}K=M_{2},$$

and solved accordingly as $K^{*}={\left(M_{1}{}^{T}M_{1}\right)}^{-1}M_{1}^{T}M_{2}$.

**Figure 5:**
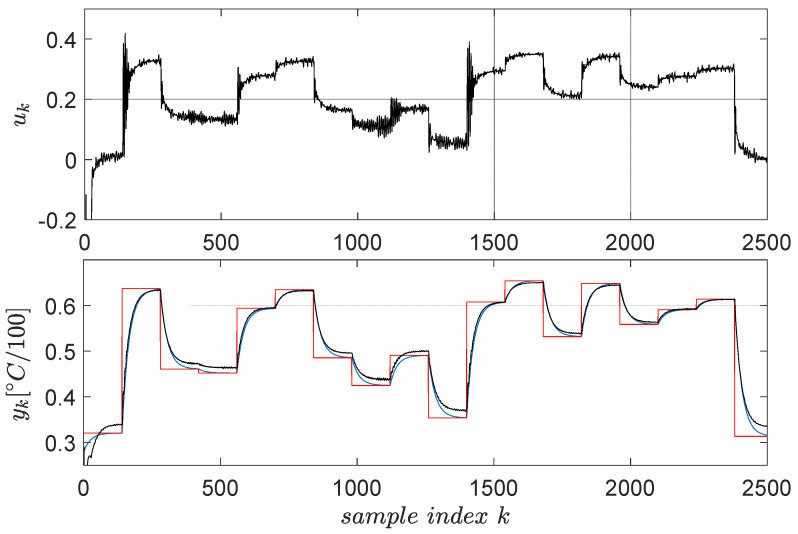
ATCS control test employing the virtual state-feedback linear compensator $K^{*}$. The red line is the reference input and the blue line is the reference model’s output, whereas the actual CLCS’s output $y_{k}$ is in black.

Following the previous steps, the linear virtual state-feedback compensator matrix is $K^{*}={\left[-8.532,-1.9441,\;9.8722,\;0.5199,\;0.4131,\;1.0127\right]}^{T}\in {ℜ}^{6}$, where the fifth value 0.4131 represents the offset gain. Testing the controller in closed loop shows the behavior in Figure 5.

A satisfactory model reference tracking performance is achieved, as seen in Figure 5, thus ensuring indirect CLCS feedback linearization. The VSFRT controller is only linear; however, nonlinear structures such as NNs have intensively been employed [2,4]. Uniformly ultimately bounded (UUB) stability of the CLCS with the proposed nonlinear VSFRT controllers was analyzed according to Theorem 1 and Corollary 1 from [4]. The learned process is also one-shot, and no iterations are performed similarly to other learning paradigms such as value iteration reinforcement Q-learning [1,3,5,53].

### 3.3. Intermediate L2 Level Primitives Learning with EDMFILC

The closed-loop feedback control system is treated as a linear dynamical system from the reference input $\rho_{k}$ to the controlled output $y_{k}$. To apply the primitive-based prediction mechanism for high-performance tracking without learning by repetitions, the primitives (the pairs of reference inputs–controlled outputs) must be learned in the first instance. The reason is that the primitive outputs must describe a shape having good approximation capacity (e.g., a Gaussian shape or others, according to the function approximation theory). This is enabled by employing the EDMFILC theory to learn such primitive pairs by trials/iterations/repetitions. This is only achieved once, to populate the library of primitives, after which the optimized reference input prediction does not require relearning by repetition.

The EDMFILC theory has been developed for linear multi-input, multi-output (MIMO) systems [1,2,3]. In the case of the SISO ATCS, the EDMFILC is particularized as follows.

Let the ATCS closed-loop reference input at the current iteration be defined, in lifted (or super-vectorial) notation spanning an *N*-samples experiment, as $R^{j}={\left[\rho_{1}^{j},\;\rho_{2}^{j},\ldots ,\;\rho_{N}^{j}\right]}^{T}\;\in {\mathbb{R}}^{N\times 1}$, where $\rho_{k}^{j}$ is the *k*th sample from the reference input of iteration j. Similarly, define $Y^{j}={\left[y_{1}^{j},\;y_{2}^{j},\ldots ,\;y_{N}^{j}\right]}^{T}\;\in {\mathbb{R}}^{N\times 1}$, where $y_{k}^{j}$ is the *k*th sample from the ATCS’s output at iteration j. The iteration–invariant desired trajectory is defined as $Y^{d}={\left[y_{1}^{d},\;y_{2}^{d},\ldots ,\;y_{N}^{d}\right]}^{T}\;\in {\mathbb{R}}^{N\times 1}$, where $y_{k}^{d}$ is the *k*th sample from the desired output, constant for all iterations. Additionally, $E^{j}={\left[e_{1}^{j}=y_{1}^{j}-y_{1}^{d},\ldots ,\;e_{N}^{j}=y_{N}^{j}-y_{N}^{d}\right]}^{T}\;\in {\mathbb{R}}^{N\times 1}$, where $e_{k}^{j}$ is the *k*th sample of the output tracking error at iteration j. Non-zero initial conditions, delays, offsets and non-minimum-phase responses must be properly considered when defining the desired trajectory.

The optimal reference input $\rho_{k}^{*}$ ($R^{*}$ in lifted notation) ensuring zero tracking error is iteratively searched, using the gradient descent update law

(10) $$R_{}^{j+1}=R_{}^{j}-\chi \frac{\partial J\left(R^{j}\right)}{\partial R},$$

where χ is the positive definite learning gain and $\frac{\partial J\left(R^{j}\right)}{\partial R}$ is the gradient of the cost function $J\left(R\right)=\frac{1}{N}\|E\left(R\right){\|}_{2}^{2}$ with respect to its argument R, evaluated at the current iteration reference input vector $R^{j}$. This cost function penalizes the tracking error over the entire trial. For linear systems, the gradient is experimentally obtainable in a model-free manner, as shown by the application steps of the EDMFILC [3]:

*Step* 1. $R^{0}=Y^{d}$ is the initialized reference input. With each iteration j, follow the next steps.

*Step* 2. Set $R^{j}$ as reference input to the closed-loop ATCS and record the current iteration tracking error $E^{j}=Y^{j}-Y^{d}$. Let this be the nominal experiment.

*Step* 3. Upside-down flip $E^{j}$ to result in $udf\left(E^{j}\right)$.

*Step* 4. Scale $udf\left(E^{j}\right)$ in amplitude by the scalar multiplication gain μ.

*Step* 5. Use $\mu \cdot udf\left(E^{j}\right)$ as an additive disturbance for the current iteration reference $R^{j}$. Use $R^{j}+\mu \cdot udf\left(E^{j}\right)$ as the reference input to what is called “the gradient experiment” and record the output $Y_{G}^{j}$ from this non-nominal experiment.

*Step* 6. As shown in [3], the gradient in (10) is computable as $\frac{\partial J\left(P^{j}\right)}{\partial P}=\frac{2}{N}\cdot udf\left(1/\mu \cdot \left(Y_{G}^{j}-Y^{j}\right)\right)$.

*Step* 7. Update $R^{j}$ based on (10).

*Step* 8. Repeat from Step 2 until the maximum number of iterations is reached or the gradient norm $\|\frac{\partial J\left(R^{j}\right)}{\partial R}{\|}_{2}$ is below some predefined threshold.

After *Step* 8, the learned primitive $\left\{R^{j},Y^{j}\right\}$ is stored in the library as $\left\{R^{\left[m\right]},Y^{\left[m\right]}\right\}$, with m indexing the *m*th primitive. Here,$\;R^{\left[m\right]}$ is the *m*th primitive input, whereas$\;Y^{\left[m\right]}$ is the *m*th primitive output.

The choice of the learning gain factor χ was proposed in [1,2,3], such that it ensures safe learning convergence. The reference input and the controlled output data from the closed-loop test of Figure 5 allow for the identification of a linear output-error (OE) approximation model T(q) of the closed-loop ATCS. Furthermore, M(q) is another good approximation model of the closed-loop ATCS, resulting via the model reference matching solved via VSFRT. Then, we solve

(11) $$\chi_{1}^{*}=arg\underset{\chi_{1}\;}{max}\;\chi_{1},s.t.\;\chi_{1}>0,\;\|1-\frac{2}{N}T\left(q\right)\chi_{1}T\left(\frac{1}{q}\right){\|}_{\infty }<1,\;\chi_{2}^{*}=arg\underset{\chi_{2}}{max}\;\chi_{2},s.t.\;\chi_{2}>0,\;\|1-\frac{2}{N}M\left(q\right)\chi_{2}M\left(\frac{1}{q}\right){\|}_{\infty }<1,\;\chi^{*}=min\left(\chi_{1},\chi_{2}\right),$$

to obtain the value $\chi^{*}=99.76$ which, for the closed-loop ATCS, is the most conservative learning gain that ensures zero tracking error in the long-term iteration domain, when applying EDMFILC.

For the ATCS, two primitives are learned by EDMFILC. Experiments are performed in a room with a controlled temperature environment, ensuring strong repeatability. The first primitive is defined by the desired trajectory $y_{k}^{d}=0.4+0.2e^{-{\left(k\cdot T_{s}-1000\right)}^{2}/50000},\;1\leq k\leq 100$, having Gaussian shape and lasting for 2000 s in the 20-s sampling period. The factor 0.4 defines the operating point (corresponding to $40^{\;\circ }C$), the factor 0.2 sets the Gaussian height, the factor 1000 sets the Gaussian center and the factor 50,000 sets its time-width. The scaling factor for the upside-down flipped error in the gradient experiment is μ=3, for a maximum of 40 EDMFILC iterations.

**Figure 6:**
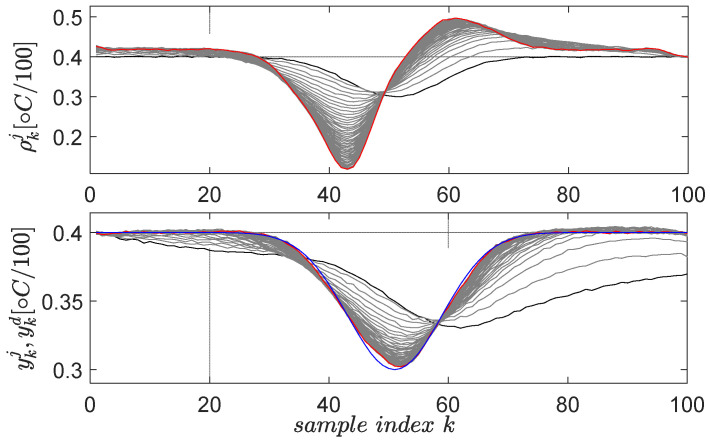
The second learned primitive: initial reference input $\rho_{k}^{0}$ and corresponding output $y_{k}^{0}$ are in black lines; the desired trajectory $y_{k}^{d}$ is in blue; the final $\rho_{k}^{30}$ and $y_{k}^{30}$ are in red. The intermediate trajectories throughout the learning phase are in grey.

The second learned primitive is defined by the desired trajectory $y_{k}^{d}=0.4-0.1e^{-{\left(k\cdot T_{s}-1000\right)}^{2}/50000},\;1\leq k\leq 100$, again having a Gaussian shape, but this time pointing downwards. All the parameters preserve the same interpretation from the first primitive. The learning gain and the scaling factor are the same. The resulting learning history is shown in Figure 6 for 30 iterations.

From the implementation viewpoint, each trial requires repeatability of the initial conditions, i.e., to reach the vicinity of the initial temperature of the desired profile $y_{k}^{d}$, after which, the data logging starts. Although the closed-loop ATCS is not perfectly linear, but rather, smooth and nonlinear, the EDMFILC is applicable and robust to such behavior. It is a pure data-driven technique relying on input–output data to learn trajectory tracking by repetitions/trials. The resulting primitive pairs are $\left\{R_{}^{\left[1\right]},Y^{\left[1\right]}\right\}$ and $\left\{R_{}^{\left[2\right]},Y^{\left[2\right]}\right\}$ and are memorized in the primitives’ library. They are called the original primitives. Each original primitive contains the reference input and the closed-loop ATCS controlled output from the last EDMFILC iteration. Therefore, each primitive intrinsically encodes the CLCS dynamics within its signals. These encoded dynamics will be used, although not explicitly, to predict the optimal reference ensuring tracking of new desired trajectories.

### 3.4. Optimal Tracking Using Primitives at the Uppermost Level L3

The final application step of the primitive-based HLF concerns the optimal reference input prediction that ensures a new desired trajectory is tracked as accurately as possible. This has to be performed without relearning the reference inputs on a trial-by-trial basis, as it was with EDMFILC. The concept has been thoroughly described in [1,2,3].

The new desired trajectory for the ATCS is $y_{k}^{d}=\min\left\{0.5,\;\max\left\{0.3,\;0.4+0.05\cdot \sin\left(0.002kT_{s}\right)+0.00001kT_{s}\right\}\right\},\;k=\bar{1,\;400}$. This trajectory’s length is four times greater than the length of each of the two learned primitives (lasting for 100 samples each). A linear regression dedicated to approximation purposes is to be solved at the level L3, which is costly when the length of $y_{k}^{d}$ is large. The strategy is to divide $y_{k}^{d}$ into shorter segments (herein four), then predict and execute the tracking on each resulting segment (or subinterval). The first segment is the part of $y_{k}^{d}$ corresponding to 1≤k≤100, consisting of N=100 samples. However, the length of a segment does not have to equal that of a primitive, although it should be about the same order of magnitude. After predicting the optimal reference for this first segment, the next segment from $y_{k}^{d}$ is extracted, the optimal reference input for its tracking is predicted, and so on until $y_{k}^{d}$ is entirely processed.

Therefore, the discussion about how to predict the optimal reference input is detailed for a single segment. For such a segment of length N (assumed even without generality loss), let the desired trajectory in lifted notation be $Y^{d}\in {\mathbb{R}}^{N\times 1}$. The steps enumerated below are performed.

*Step* 1. Extend $Y^{d}$ to length 2N to result in $Y^{d\left[e\right]}\in {\mathbb{R}}^{2N\times 1}$, by padding the leftmost N/2 samples having the value of the first sample from $y_{k}^{d}$ and the rightmost N/2 samples having the value of the last sample from $y_{k}^{d}$.

*Step* 2. Each original primitive $\{R_{}^{\left[\delta \right]},Y^{\left[\delta \right]}\},\delta \in \left\{1,2\right\}$ is extended by the same principle, with padding to length 2N as performed for $Y^{d}$. The resulting extended primitives are $\{R_{}^{\left[\delta e\right]},Y^{\left[\delta e\right]}\},\delta \in \left\{1,2\right\}$, where each signal $R_{}^{\left[\delta e\right]},Y^{\left[\delta e\right]}$ (here expressed in lifted form) has length 2N.

*Step* 3. A number of M random copies of the extended primitives are memorized. These copies are themselves primitives, indexed as $\{R_{}^{\left[\pi e\right]},Y^{\left[\pi e\right]}\},\pi =\bar{1,M}$.

*Step* 4. Each copied primitive is delayed/advanced by an integer uniform value θ∈[−N,N]. The delayed copies are indexed as $\{R_{}^{\left[\theta_{\pi }e\right]},Y^{\left[\theta_{\pi }e\right]}\},\pi =\bar{1,M}$. Padding has to be used again because the delay is without circular shifting. To this extent, a number of |θ| samples will be padded with the value of the first or last unshifted samples from $R_{}^{\left[\pi e\right]}$ and $Y^{\left[\pi e\right]}$, respectively.

*Step* 5. An output basis function matrix is built from the delayed primitive outputs as $ℬ=\left[Y_{}^{\left[\theta_{1}e\right]},\ldots ,Y_{}^{\left[\theta_{M}e\right]}\right]\in {\mathbb{R}}^{2N\times M}$. These columns correspond to Gaussian signals which were extended, delayed and padded as indicated in the previous steps. The columns of ℬ serve as approximation functions for the extended and padded desired trajectory $Y^{d\left[e\right]}$, by linearly combining them as $ℬ\beta ,\;\beta ={\left[\beta_{1},\ldots ,\beta_{M}\right]}^{T}\in {\mathbb{R}}^{M}$.

*Step* 6. Find the optimal $\beta^{*}=\arg\underset{\beta }{\min}\|ℬ\beta -Y^{d\left[e\right]}{\|}_{2}^{2}$ by solving the overdetermined linear equation system with least squares.

*Step* 7. Employing the linear systems superposition principle in order to obtain the optimal reference input leading to optimal tracking of $Y^{d\left[e\right]}$, we compute $R_{}^{*\left[e\right]}=\overset{M}{\underset{\pi =1}{\sum }}\beta_{\pi }R_{}^{\left[\theta_{\pi }e\right]}$. Here, $R_{}^{*\left[e\right]}\in {\mathbb{R}}^{2N}$, and it was shown in Theorem 1 from [3] that $R_{}^{*\left[e\right]}$ theoretically ensures the smallest tracking error, only bounded by the approximation error of the difference $Y_{}^{\left[b\right]}\beta -Y^{d\left[e\right]}$.

*Step* 8. To return to N-length signals, the true reference input $R_{}^{*}$ is obtained by clipping the middle interval of $R_{}^{*\left[e\right]}$. The signal $R_{}^{*}$ ($\rho_{k}^{*}$ in time-based notation) is the predicted optimal reference input which is to be set as reference input to the closed-loop ATCS, to execute the tracking task on the current segment.

For the application of the previous steps for the ATCS, we used a number of M=2400 copies of the original two primitives, with corresponding delays are uniformly random integers within [−100;100], hence spanning 200 samples for extended trajectories of 200 samples.

A secondary aspect is the constraint satisfaction being addressed by the proposed primitive-based learning framework. As discussed in [1,2,3], the straightforward approach to indirectly address controlled output magnitude constraints is to enforce magnitude constraints upon the new trajectory $y_{k}^{d}$. In this case, the role of the max,min operators used within the definition of $y_{k}^{d}$ is to enforce such constraints by trajectory magnitude clipping. This is a form of soft-constraint handling [3]; the accuracy is evaluated only after executing the tracking task and may vary. Other types of constraints, such as rate constraints, may be handled similarly, in the presented indirect style. Constraints on other CLCS characteristic signals are not considered as relevant, be it magnitude or rate inequality constraints: the ones on the CLCS’s inputs are too “embedded” and they negatively influence the model matching achievement, whereas the ones on the reference input again affect the trajectory tracking accuracy at the CLCS’s output.

**Figure 7:**
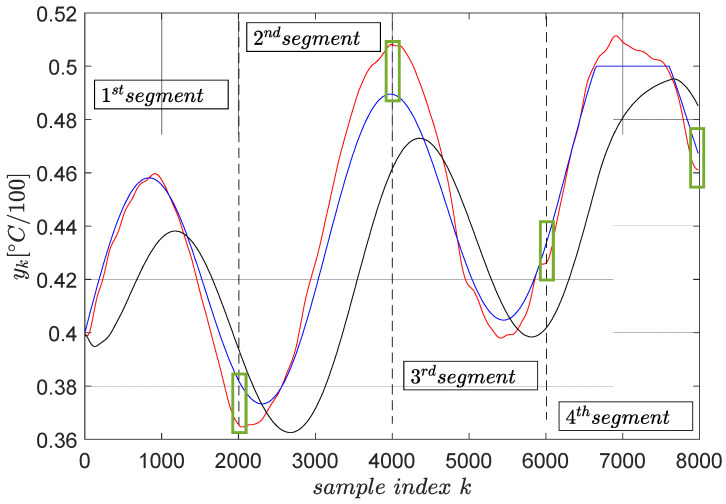
The final trajectory tracking results of $y_{k}^{d}$ (blue). The output $y_{k}$ (red) when the optimal reference input ($\rho_{k}^{*}$ from $R^{*}$) is computed using primitives and the output $y_{k}$ (black) when the reference input is $\rho_{k}=y_{k}^{d}$. The green boxes highlight the tracking errors at the end of each segment.

The trajectory tracking results on a segment-by-segment basis is shown in Figure 7.

Several remarks are given. The tracking accuracy is expressed as the cumulated tracking error squared norm divided by the number of samples, which is the common mean summed squared error. The MSE obtained with the reference input is optimally predicted based on the primitive approach measures $4.75\times 10^{-4}$. The same indicator measured when the reference input is $\rho_{k}=y_{k}^{d}$ is $1.36\times 10^{-3}$, nearly three times larger. This clearly shows that the primitive-based approach effectively achieves higher tracking accuracy. Its anticipatory character is revealed in the sense that the noncausal filtering operations involved lead to a reference input which makes the CLCS respond immediately when the desired trajectory changes. Therefore, it eliminates the lagged response of the naturally low-pass CLCS.

Furthermore, the green boxes in Figure 7 highlight the tracking errors at the end time of the tracking execution on each segment. These errors do not build up, being regarded as non-zero initial conditions for the next segment tracking task, with their effect vanishing in time.

Constraint condition imposed on the upper-clipped magnitude of the desired trajectory in the fourth segment does not reflect very accurately upon the controlled output. The cause of this, as well as the only three-fold improvement in the tracking accuracy with the primitive-based approach, is due to the CLCS not being so linear (not perfectly matching the reference model M(q)). The importance of achieving high-quality model reference matching (and therefore indirect closed-loop linearization) was identified as crucial [3]. When the linearity assumption holds, the accuracy may be improved up to 100-fold, and the output constraints are thoroughly enforced. This has been reported in other applications [1,2,3]. Therefore, ensuring the low-level model reference matching (or tracking) of the CLCS is critical.

## 4. The Electrical Braking System (EBS)

### 4.1. System Description

**Figure 8:**
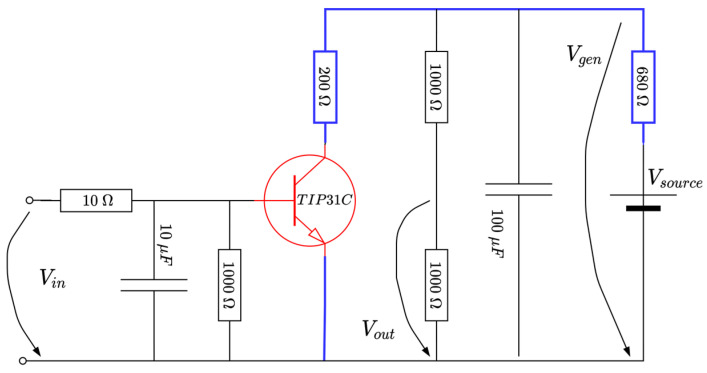
Schematic diagram of the EBS (adapted from [58]).

A rheostatic brake emulator is next considered as a representative case study (please refer to Figure 8 below). Such a process has wide applicability in resistive-based braking in cars, trains or in wind turbine generators [58]. Suppose there exists a variable voltage source $V_{source}$ (due to irrelevant conditions), the goal would be to regulate a constant voltage $V_{gen}$ across a section of the circuit, to ensure, e.g., a constant power delivery over some load. In practice, the (resistive) load consumer is changed accordingly, to adjust for the voltage level. By Ohm’s law, keeping a constant load while changing the current achieves an equivalent effect. Hence, the means to control $V_{gen}$ is achieved by current variation through the blue line in Figure 8, achievable by changing the current flow through a (power) transistor (indicated on the blue path in the figure). From a practical perspective, however, it is easier to maintain $V_{source}$ at a constant level and instead control the voltage level $V_{gen}$, to basically illustrate the same effect.

Some technical facts about the circuit are detailed. The source voltage is 9 V, a TIPC31C power transistor is used (capable of up to 40 W in switching operation mode, colored red in Figure 8). The transistor’s base voltage is compatible with the voltage level obtained from the PWM output of an Arduino board, in this case represented by $V_{in}\in \left[0;5\right]V$. Therefore, the actual control input $u_{k}$ will be the duty cycle to the variation in $V_{in}$ within its domain. MATLAB software-side processing uses the values {0,…,255} to write the PWM port; thus, the equation to derive the voltage $V_{in}$ as a function of $u_{k}$ is

(12) $$V_{in}\left[V\right]=\frac{5}{255}\times sat_{0}^{255}\left\{255-\frac{255}{100}sat_{0}^{100}\left(30+100u_{k}\right)\right\},$$

where the operator $sat_{L}^{H}\left(.\right)$ saturates its argument within [L;H] values and the value “30” is offset to ensure a voltage of $V_{in}=3.5\;V$ at the transistor’s base, around the linear operating point. Therefore, if $u_{k}$ increases, the $V_{in}$ decreases, the transistor opens (starts acting as open-switch), the current decreases and $V_{gen}$ also increases, whereas vice versa holds. For our case, the domain of the non-dimensional duty cycle factor $u_{k}$ is [0;1].

**Figure 9:**
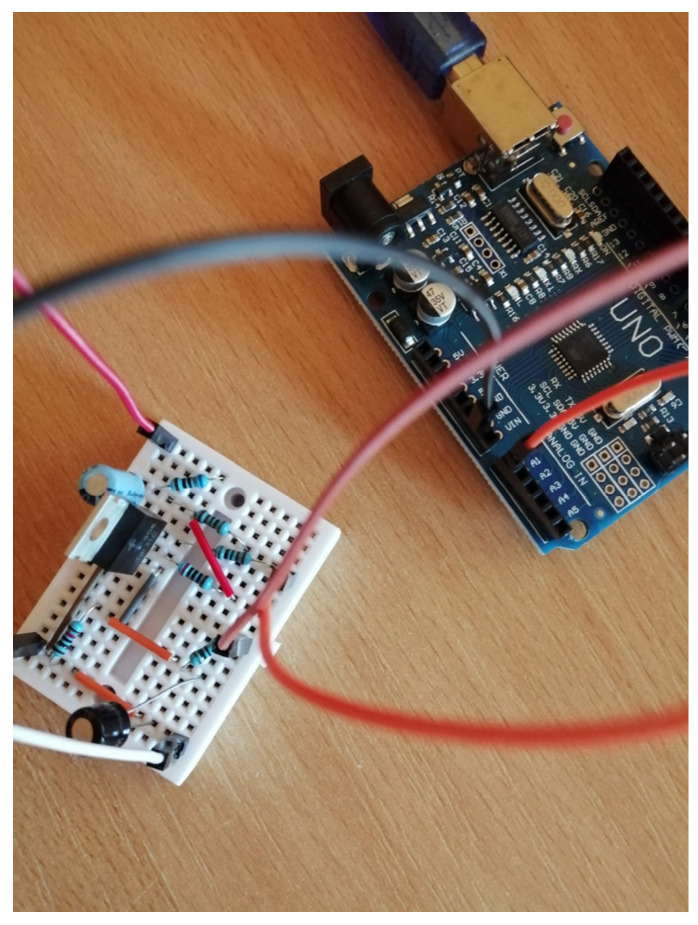
Illustration of the hardware realization of the EBS.

The voltage $V_{in}$ is low-pass-filtered through an RC stage made up of a 10 kΩ resistor and 10 μF capacitor. Therefore, the resulting filtered output will actually drive the TIP31C transistor in its linear operation mode. Additional stage elements use a 100 μF capacitor to clean $V_{source}$ from noise and a voltage divider to reduce $V_{gen}$ to $V_{out}$ to within the voltage levels [0;5] V acceptable for the ADC input Arduino port. The resulting voltage level $V_{out}$ is software-processed, multiplied by two and filtered through 1/(0.2s+1) to recover the original value $V_{gen}$. For the given $V_{source}=9\;V$ and the other electrical components, the effect is that the controlled output is $y_{k}=V_{gen}\in \left[2;6.7\right]\;V$, whose level is to be controlled within its entire domain. The sampling period $T_{s}=0.05$ s is suitable for data acquisition and control inference. A picture of the realized EBS hardware attached to the Arduino board is rendered in Figure 9. The system response is rather fast and subject to noise, making it challenging for all control stages. Importantly, the EBS module is fairly cheap and can be used by many practitioners.

### 4.2. EBS Input–Output Data Collection for Learning Low-Level L1 Control Dedicated to Model Reference Tracking

**Figure 10:**
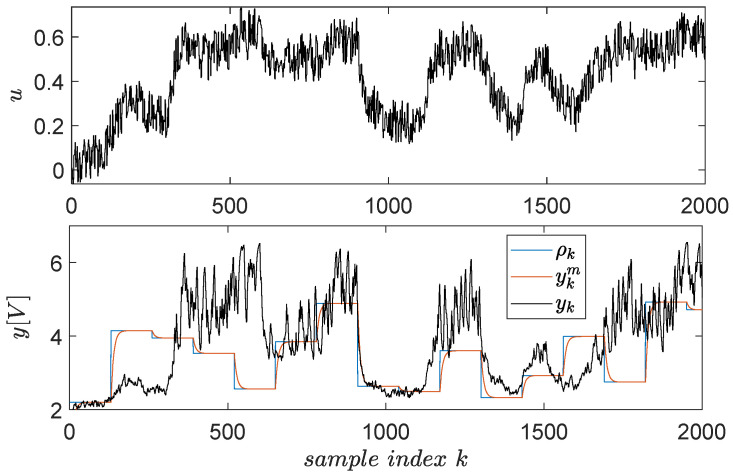
Input–output data samples gathered from the EBS.

A dataset of input–output samples is measured from the EBS in the first place, to learn the level L1 controller. Exploration quality is important because it stimulates all system dynamics [58,59]. Long experiments ensure that many combinations of $u_{k}$ and $y_{k}$ are visited; however, it is of interest to accelerate the collection phase, i.e., to obtain more variation from the signals in the same unit of time. This can only be obtained with the help of a closed-loop controller which compensates the system’s dynamics, ensuring faster transients. To this extent, a discrete-time version of the integral-type controller C(s)=1/s is used. The reference input driving the CLCS over the EBS is a staircase signal switching amplitude at every five seconds and whose amplitudes are uniformly random values in [2.2;6]V. Additive stair-like noise perturbs the reference input, with a shorter switching period of 0.1 s and uniform random amplitudes within [−0.1;0.1]. The noise’s role is to further break the time correlation between successive samples. The resulting input–output explored data depicted in Figure 10.

VSFRT is used again for learning a virtual state feedback controller. The reference model M(q) is the discretized variant of M(s)=1/(0.4s+1) at 0.05 s. This selection correlates strongly with the EBS’s open-loop bandwidth, no observed time-delay, no open-loop step response overshoot and minimum-phase type.

A nonlinear long short-term-memory (LSTM) recurrent neural network controller $u_{k}=C\left(s_{k}^{ext}\right)$ is learned as suggested in [12], based on the input–output controller data sequence $\left\{\left(s_{k}^{ext}≜{\left[{\tilde{s}}_{k}^{T},{\tilde{\rho }}_{k}\right]}^{T},u_{k}\right)\right\}$ which is calculated offline according to the VSFRT principle, after measuring $\left\{u_{k},\;y_{k}\right\}$. The controller’s LSTM network is modelled in discrete time, based on the LSTM cell as

(13) $$i_{k}=logsig\left(W_{i}s_{k}^{ext}+R_{i}h_{k-1}+b_{i}\right),\;f_{k}=logsig\left(W_{f}s_{k}^{ext}+R_{f}h_{k-1}+b_{f}\right),\;\;g_{k}=tanh\left(W_{g}s_{k}^{ext}+R_{g}h_{k-1}+b_{g}\right),\;o_{k}=logsig\left(W_{o}s_{k}^{ext}+R_{o}h_{k-1}+b_{o}\right),\;\mathrm{where}\;c_{k}=f_{k}⨂c_{k-1}+i_{k}⨂g_{k},\;h_{k}=o_{k}\;⨂\;tanh(c_{k}),\;y_{k}^{LSTM}=W_{y}h_{k}+b_{y},$$

where logsig(.) is the sigmoid function applied element-wise, tanh(.) is the hyperbolic tangent applied element-wise, ⨂ multiplies vectors element-wise, $h_{k}\in {\mathbb{R}}^{n_{h}}$ is the hidden LSTM state of size $n_{h}$ at time step k, $c_{k}\in {\mathbb{R}}^{n_{h}}$ is the LSTM cell state at step k, $s_{k}^{ext}\in {\mathbb{R}}^{\xi }$ is the exogeneous input sequence of size ξ, $i_{k}\in {\mathbb{R}}^{n_{h}}$ is from the input gate, $f_{k}\in {\mathbb{R}}^{n_{h}}$ is from the forget gate, $g_{k}\in {\mathbb{R}}^{n_{h}}$ is the cell candidate and $o_{k}\in {\mathbb{R}}^{n_{h}}$ is from the output gate. $W_{j}\in {\mathbb{R}}^{n_{h}\times \xi },j\in \left\{i,f,g,o\right\}$ are the cell input weights, $R_{j}\in {\mathbb{R}}^{n_{h}\times n_{h}},j\in \left\{i,f,g,o\right\}$ are the cell recurrent weights and $b_{j}\in {\mathbb{R}}^{n_{h}\times 1},j\in \left\{i,f,g,o\right\}$ are the cell offsets. The LSTM network output is $y_{k}^{LSTM}\in {\mathbb{R}}^{n_{LSTM}}$ and linearly depends on $h_{k}$ through the network output weights $W_{y}\in {\mathbb{R}}^{n_{LSTM}\times n_{h}},\;b_{y}\in {\mathbb{R}}^{n_{LSTM}\times 1}$. Here, $\pi =\left\{W_{j},R_{j},b_{j},W_{y},b_{y}\right\},j\in \left\{i,f,g,o\right\}$ collates all the trainable elements of the LSTM network.

The cell input weights are initialized with Xavier algorithm, the cell recurrent weights are initialized orthogonally, and the offsets are all zero except for the $b_{f}$ which are set to one. $W_{y}$ is also initialized with Xavier, whereas $b_{y}$ are all zero at first. The Adam algorithm is used for training for a maximum of 1000 epochs, over minibatches of 64 elements, initial learning rate is 0.01, gradient clipping threshold is 5, and 80% of the dataset is used for training; the remaining 20% is for validation after each 10 epochs. The loss training is the mean squared error (MSE) with $L_{2}$ weights regularization factor of $10^{-4}$.

The VSFRT LSTM-based recurrent neural network controller is found after the next steps are applied in order [58].

*Step* 1. Define the observability index τ=3 and construct the trajectory $\left\{u_{k},\;y_{k},{\tilde{s}}_{k}\right\}$, where ${\tilde{s}}_{k}={\left[y_{k},\;y_{k-1},\;y_{k-2},\;y_{k-3},\;u_{k-1},\;u_{k-2},\;u_{k-3}\;\right]}^{T}\in {\mathbb{R}}^{7}$ is a virtual state for EBS. Using the discrete time index $k=\bar{1,N}$, 1997 tuples of the form $\left\{u_{k},\;y_{k},{\tilde{s}}_{k}\right\}$ are built.

*Step* 2. The virtual reference input is obtained as ${\tilde{\rho }}_{k}=M^{-1}\left(q\right)y_{k}^{f}$, where $y_{k}^{f}$ is the low-pass-filtered version of $y_{k}$ through the filter $\frac{0.1}{1-0.9q^{-1}}$, due to $y_{k}$ being noisy.

*Step* 3. Construct the regressor state as $s_{k}^{ext}={\left[{\tilde{s}}_{k}^{T},\;{\tilde{\rho }}_{k}\right]}^{T},1\leq k\leq 1997$.

*Step* 4. Parameterize the LSTM-based VSFRT controller $u_{k}=C\left(s_{k}^{ext},\pi \right)$ with $\xi =8,n_{h}=10,\;n_{LSTM}=1$. Initialize the controller network parameters according to the settings. The VSFRT goal is to ensure model reference matching by indirectly solving the controller identification problem [1,4,12,53]

(14) $$\pi^{*}=arg\underset{\pi }{min}V_{VR}^{N}\left(\pi \right),\;V_{VR}^{N}\left(\pi \right)=\frac{1}{N}\sum_{k=1}^{N}{\left|\left|u_{k}-C\left(s_{k}^{ext},\pi \right)\right|\right|}^{2},$$

which, in fact, means training the LSTM network with input sequences $\left\{s_{k}^{ext}\right\}$ and output sequences $\left\{u_{k}\right\}$ in order to minimize the mean squared prediction errors with weight regularization.

**Figure 11:**
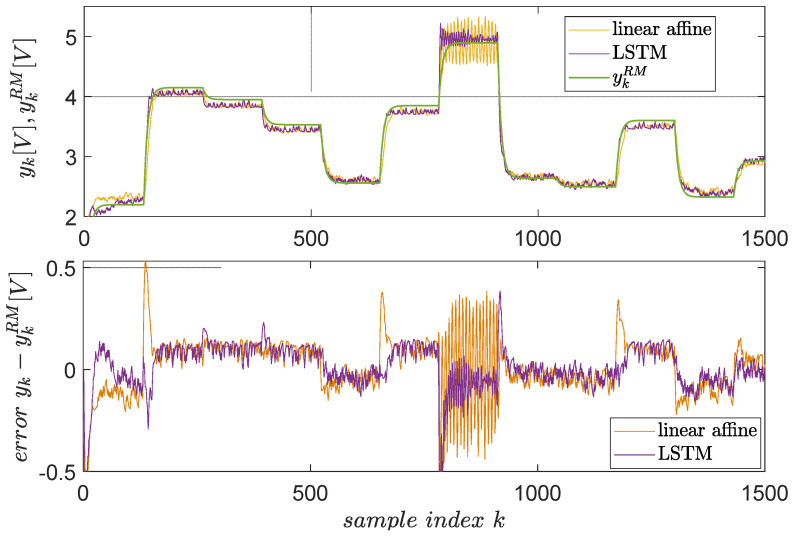
Closed-loop control test for EBS, with the linear affine VSFRT controller from [58] of compensator $K^{*}$ vs. the proposed LSTM-based controller. The reference model’s output is green, the actual closed-loop $y_{k}$ is orange with the linear affine controller and magenta with the LSTM controller.

Following the previous steps, the resulting LSTM-based VSFRT controller is tested in a closed-loop against a linear-affine VSFRT controller $u_{k}=K^{T}s_{k}^{ext}$ (but this time with $s_{k}^{ext}$ including an extra feature “1” to model the affine term), as learned in [58]. The results are shown in Figure 11. The superiority of the nonlinear, recurrent LSTM controller is clear in terms of smaller errors and fewer oscillations at higher setpoint values where EBS changes its character. The reason is that LSTM is better for learning long-term dependencies from time series.

Subsequently, the EBS closed-loop is considered to be sufficiently linearized to match M(q); hence, the level L2 learning phase is next attempted.

### 4.3. Intermediate L2 Level Primitives Learning with EDMFILC

For the EBS, the two primitives are learned by the same EDMFILC procedure that was also applied for the ATCS.

**Figure 12:**
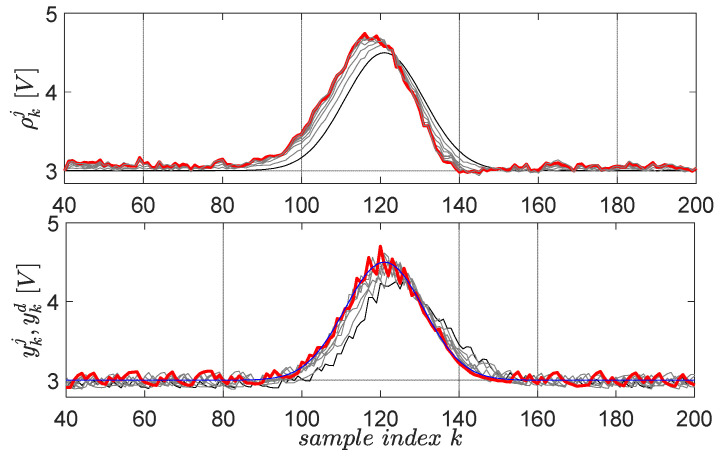
The first EBS learned primitive pair: initial reference input $\rho_{k}^{0}$ and corresponding output $y_{k}^{0}$ are in black lines; the desired trajectory $y_{k}^{d}$ is in blue; the final $\rho_{k}^{10}$ and $y_{k}^{10}$ are in red. The intermediate trajectories throughout the learning process are in grey.

The first primitive is defined by the desired trajectory $y_{k}^{d}=\zeta_{1}+\zeta_{2}e^{-{\left(k\cdot T_{s}-\zeta_{3}\right)}^{2}/\zeta_{4}},\;1\leq k\leq 200$, having Gaussian shape and lasting for 8 s in the 0.05-s sampling period, starting after 2 s in which the system closed-loop system stabilizes its output at 3V [59]. The factor $\zeta_{1}=3$ defines the 3 V operating point offset level, the factor $\zeta_{2}=1.5$ sets the Gaussian magnitude, the factor $\zeta_{3}=6$ fixes the Gaussian center and the factor $\zeta_{4}=0.5$ fixes its time-width. The scaling factor for the upside-down flipped error in the gradient experiment is μ=3, for a maximum of 10 EDMFILC iterations. The learning gain is safely chosen as $\chi^{*}=153.7$ based on the procedure Erom Equation (11) (using M(q) and an identified OE first-order model $0.1227q^{-1}/\left(1-0.8797q^{-1}\right)$), in order to guarantee learning convergence [3]. The learning process of the first primitive is shown in Figure 12.

**Figure 13:**
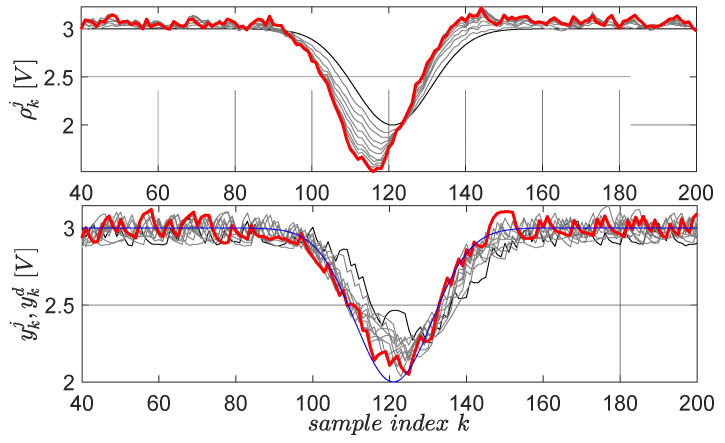
The second EBS learned primitive pair: initial reference input $\rho_{k}^{0}$ and corresponding output $y_{k}^{0}$ are in black lines; the desired trajectory $y_{k}^{d}$ is in blue; the final $\rho_{k}^{10}$ and $y_{k}^{10}$ are in red. The intermediate trajectories throughout the learning process are in grey.

The second learned primitive is defined by the desired trajectory $y_{k}^{d}=\zeta_{1}-\zeta_{2}e^{-{\left(k\cdot T_{s}-\zeta_{3}\right)}^{2}/\zeta_{4}},\;1\leq k\leq 200$, ($\zeta_{1}=3,\zeta_{2}=1,\zeta_{3}=6,\zeta_{4}=0.5$), again having a Gaussian shape, but this time pointing downwards. All the parameters preserve the same interpretation from the first primitive. The learning gain and the scaling factor are the same. The resulting learning history is shown in Figure 13 for 10 iterations.

From a practical validation viewpoint, all normal and gradient EDMFILC experiments start when the controlled voltage output reaches 3V, which is the operating point. For this reason, only N=160 samples are actual primitives (Figure 12 and Figure 13), and the first 40 samples out of the 200 allow for the EBS closed-loop to reach the operating point.

Although challenging, the noisy closed-loop EBS is capable of learning the primitives under the linearity assumption about the closed-loop, even in a low signal-to-noise ratio environment.

The resulting original primitives are $\left\{R_{}^{\left[1\right]},Y^{\left[1\right]}\right\}$ and $\left\{R_{}^{\left[2\right]},Y^{\left[2\right]}\right\}$ and memorized in the primitive’s library. Each original primitive contains the reference input and the closed-loop EBS controlled output measured at the last EDMFILC iteration. In the next step, the final level L3 learning occurs, where the original primitives will be used to predict the optimal reference input which ensures that a new desired trajectory is optimally tracked, without having to relearn tracking by EDMFILC trials.

### 4.4. Optimal Tracking Using Primitives at the Uppermost Level L3

The new desired trajectory at the EBS’s output $y_{k}^{d}=\min\left\{4,\;\max\left\{2.5,\;3+\sin\left(1.8kT_{s}\right)+0.05kT_{s}\right\}\right\}$ when $k=\bar{1,\;640}$, which is right-shifted with the left padding of a value of 3 for the first 40 samples. Then, $y_{k}^{d}$ is four times greater than the length of each of the two learned primitives (each one has 160 samples). The strategy is to divide $y_{k}^{d}$ into four segments and predict and execute the tracking on each resulting segment (or subinterval). The first segment is the part of $y_{k}^{d}$ corresponding to 1≤k≤160, consisting of N=160 samples. After predicting the optimal reference for this first segment, it will be set as reference input to the closed-loop EBS, and the trajectory tracking is performed. At the end, the next segment from $y_{k}^{d}$ is extracted, its corresponding optimal reference input is predicted and fed to the closed-loop, tracking is executed, etc.

The approach is similar to the first case study of the ATCS. The subsequent steps done for the EBS are performed in this order [59]:

*Step* 1. Extend $Y^{d}$ (lifted notation of $y_{k}^{d}$) to length 2N (320 samples in this case) to get $Y^{d\left[e\right]}\in {\mathbb{R}}^{2N\times 1}$, by padding the leftmost N/2 samples with the value of the first sample of $y_{k}^{d}$ and the rightmost N/2 samples with the value of the last sample from $y_{k}^{d}$.

*Step* 2. Each of the original primitives $\{R_{}^{\left[\delta \right]},Y^{\left[\delta \right]}\},\delta \in \left\{1,2\right\}$ is similarly extended with padding to length 2N, as was performed with $Y^{d}$. The resulting extended primitives are $\{R_{}^{\left[\delta e\right]},Y^{\left[\delta e\right]}\},\delta \in \left\{1,\;2\right\}$, where each signal $R_{}^{\left[\delta e\right]},Y^{\left[\delta e\right]}$ (here expressed in lifted form) has a length of 2N (320 here).

*Step* 3. M=2000 random copies of the extended primitives are memorized. These copies are themselves primitives, indexed as $\{R_{}^{\left[\pi e\right]},Y^{\left[\pi e\right]}\},\pi =\bar{1,M}$.

*Step* 4. Each copied primitive is delayed/advanced by an integer uniform value θ∈[−N,N]. The delayed copies are indexed as $\{R_{}^{\left[\theta_{\pi }e\right]},Y^{\left[\theta_{\pi }e\right]}\},\pi =\bar{1,M}$. Padding has to be used again because the delay is without circular shifting. To this extent, a number of |θ| samples will be padded with the value of the first or last unshifted samples from $R_{}^{\left[\pi e\right]}$ and $Y^{\left[\pi e\right]}$, respectively.

*Step* 5. An output basis function matrix is built from the delayed primitive outputs as $ℬ=\left[Y_{}^{\left[\theta_{1}e\right]},\ldots ,Y_{}^{\left[\theta_{M}e\right]}\right]\in {\mathbb{R}}^{2N\times M}$. These columns correspond to Gaussian signals which were extended, delayed and padded, as indicated in the previous steps. The columns of ℬ serve as approximation functions for the extended and padded desired trajectory $Y^{d\left[e\right]}$, by linearly combining them as $ℬ\beta ,\;\beta ={\left[\beta_{1},\ldots ,\beta_{M}\right]}^{T}\in {\mathbb{R}}^{M}$.

*Step* 6. The optimal weights $\beta^{*}=\arg\underset{\beta }{\min}\|ℬ\beta -Y^{d\left[e\right]}{\|}_{2}^{2}$ are found solving the overdetermined linear equation system with least squares.

*Step* 7. The reference input ensuring that $Y^{d\left[e\right]}$ is optimally tracked, computes as $R_{}^{*\left[e\right]}=\overset{M}{\underset{\pi =1}{\sum }}\beta_{\pi }^{*}R_{}^{\left[\theta_{\pi }e\right]}$. Here, $R_{}^{*\left[e\right]}\in {\mathbb{R}}^{2N}$.

*Step* 8. To return to N-length signals, the useful reference input $R_{}^{*}$ is obtained by clipping the middle interval of $R_{}^{*\left[e\right]}$. The signal $R_{}^{*}$ ($\rho_{k}^{*}$ in time-based notation) is the predicted optimal reference input which is to be set as the reference input to the closed-loop EBS, to execute the tracking task on the current segment.

The importance of dividing longer desired trajectories into segments brings lower complexity to solving for the regression coefficients $\beta^{*}$. This solves a complexity issue because the number of coefficients is in the hundreds and does not scale up well with the desired trajectory’s length (more equations added to the linear overdetermined system).

**Figure 14:**
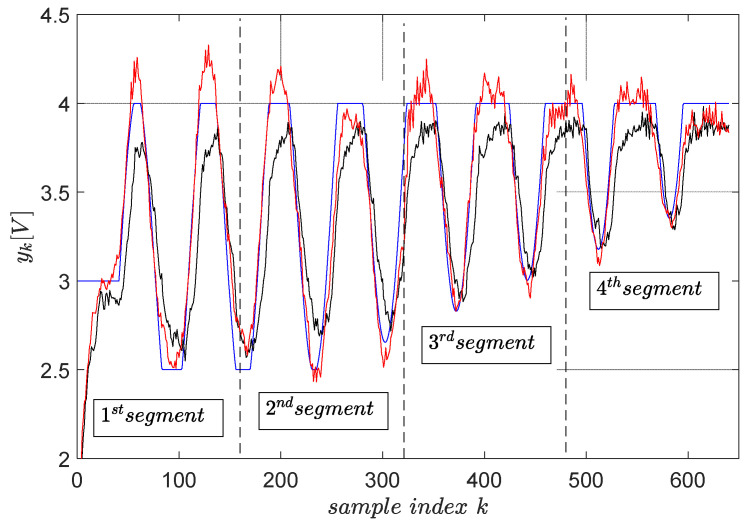
The final trajectory tracking results of $y_{k}^{d}$ (blue). The output $y_{k}$ (red) when the optimal reference input ($\rho_{k}^{*}$ from $R^{*}$) is computed using primitives and the output $y_{k}$ (black) when the reference input is $\rho_{k}=y_{k}^{d}$. The green boxes highlight the tracking errors at the end of each segment. The red and black trajectories are averaged from four runs.

Again, magnitude constraints upon the desired trajectory $y_{k}^{d}$ are enforced by clipping it with the max,min operators. The final trajectory tracking results on a segment-by-segment basis are shown in Figure 14, statistically averaged for four runs.

The tracking accuracy is measured again using the MSE index. The MSE with the reference input optimally predicted using primitives measures $5.12\times 10^{-3}$. The MSE when the reference input is $\rho_{k}=y_{k}^{d}$ measures $1.03\times 10^{-2}$, which is two times larger. This clearly shows that the primitive-based approach effectively achieves higher tracking accuracy. Its anticipatory character is revealed in the sense that the noncausal filtering operations involved lead to a reference input, which makes the CLCS respond immediately when the desired trajectory changes. Therefore, it eliminates the lagged response of the naturally low-pass CLCS.

The tracking errors at the end of each segment do not build up after the segment tracking episodes. They are seen as non-zero initial conditions for the next segment tracking task, and their effect vanishes relatively rapidly.

The upper and lower constraints imposed on the desired trajectory do not accurately transfer upon the controlled EBS output. It is still better than with the reference input being $y_{k}^{d}$. The cause of this, as well as the only two-fold improvement in the tracking accuracy with the primitive-based approach, is due to the closed-loop being not so linear (not perfectly matching the reference model M(q)). The importance of achieving high-quality model reference matching (and therefore indirect linearization of the closed-loop) is again emphasized.

## 5. Conclusions

The proposed hierarchical learning primitive-based framework has been validated on two affordable lab-scale nonlinear systems. At a low level (level L1), VSFRT was employed to learn linear-affine or nonlinear LSTM-like virtual state-feedback neuro-controllers dedicated to linear model reference matching. The learning phase relies on the nonlinearly controlled system assumed as observable, while building virtual state–space representation from present and past input–output data. Hence, VSFRT is purely data-driven and able to overcome the dynamical system’s model unavailability.

At the secondary level, EDMFILC shows resilience to smooth closed-loop nonlinearity and high amplitude noise, although being based on linearity assumptions. The ATCS and EBS are mono-variable (SISO) systems; however, EDMFILC has been shown as equally effective on multivariable (or MIMO) systems. In fact, the number of gradient experiments has been reduced to one, no matter how many control channels (complexity reduced from $O\left(N^{3}\right)$ to $O\left(N^{2}\right)$ [3]). EDMFILC should be used whenever the output primitive shape is not desirable for approximation purposes when used in the L3 phase. Hence, the purely data-driven model-free level L2 learning phase has lesser impact on the final tracking quality. The anticipative response in the final tracking response, which is due to the non-causal filtering operation, is the qualitative trait of the EDMFILC.

The uppermost L3 learning phase is based on the primitives obtained after sequentially applying the L1 and L2 learning phases. The final tracking performance and constraint satisfaction critically depend on the quality of level L1 model reference matching. To this end, most efforts should be concentrated on the level L1 successful learning.

Although the effectiveness of the primitive-based HLF has been proven, it was shown how machine learning techniques can help improve and extend the scope of the classical control systems techniques. Further validation on applications more different in nature will prove the framework’s ability to induce the CSs with some of the intelligent features of living organisms, based on memorization, learnability, generalization, adaptation and robustness.

## Data Availability

Not applicable.
